# Biomechanical Sensing Systems for Cardiac Activity Monitoring

**DOI:** 10.1155/2022/8312564

**Published:** 2022-11-18

**Authors:** Hamza Abu Owida

**Affiliations:** Medical Engineering Department, Faculty of Engineering, Al-Ahliyya Amman University, Amman 19328, Jordan

## Abstract

Cardiovascular disease is consistently ranked high among the causes of death on a global scale. Monitoring of cardiovascular signs throughout the course of a long period of time and in real time is necessary in order to discover anomalies and begin early intervention at the appropriate time. To this purpose, a significant amount of interest among researchers has been directed toward the creation of flexible sensors that may be worn or implanted and are capable of constant, immediate observation of a variety of main physiological indicators. The real-time readings of the heart and arteries' pressure fluctuations can be reflected directly by mechanical sensors, which are one of the many types of sensors. Potential benefits of mechanical sensors include excellent accuracy and considerable versatility. Capacitive, piezoresistive, piezoelectric, and triboelectric principles are the foundations of the four types of mechanical sensors that are discussed in this article as recent developments for the purpose of monitoring the cardiovascular system. The biomechanical systems that are present in the cardiovascular system are then detailed, along with their monitoring, and this includes blood and endocardial pressure, pulse wave, and heart rhythm. In conclusion, we examine the usefulness of the use of continuous health monitoring for the treatment of vascular disease and highlight the difficulties associated with its translation into clinical practice.

## 1. Introduction

Cardiovascular disease (CVD) and the complications that might arise from it are regarded as one of the primary causes of death on a global scale [1]. Furthermore, it is anticipated that this number would steadily expand to 23.6 million by the year 2030 [2]. It is also referred to as a cardiovascular disorder, which describes a collection of abnormal signs and symptoms that manifest when the body's major blood vessels fail to provide oxygen and nutrients to the brain and heart [3]. Heart attacks, strokes, and heart failure caused by poor blood flow to the heart are examples of common cardiovascular diseases [4]. They are all well known for the high rates of mortality, morbidity, and disability that they cause, and in recent years, increasingly, young adults are dominating the scene [5]. This is likely due to the fact that cardiovascular disease is caused by things like being overweight, eating poorly, not getting enough sleep, and having high blood pressure or diabetes. Enhanced medicine administration and healthcare delivery are examples of timely interventions that can result from early observation are key components in preventing the majority of deaths that are caused by cardiovascular disease [6]. The present treatment for cardiovascular disease is a process that lasts for a long time, and constant monitoring can considerably minimize the risk of cardiovascular disease returning.

Electrocardiograms (ECG), Doppler ultrasounds, arteriography, computed tomography (CT), and other imaging techniques, among others, are commonly used in clinical settings to evaluate cardiovascular disease (CVD), all of which are able to identify the disease accurately [7–9]. In addition, patients who have arrhythmia and myocardial ischemia are required to have real-time electrocardiogram monitoring [10]. Conventional cardiovascular monitoring, on the other hand, frequently has its limitations due to the paucity of medical resources, which can be inconvenient, as well as the utilization of cumbersome and pricey technology for episodic diagnosis in hospital settings.

Additionally, due to issues with durability, mobile connectivity, longevity, and reliability, it is challenging to maintain a long-term collection of clinical grade individual health indicators in traditional healthcare systems [11–13].

Consequently, to date, the need for low cost, wearable biomedical devices that reliably provide meaningful guideline health data have been a major roadblock in the promotion of the value of technology provided by the Internet of Things (IoT) in the individualized healthcare system. Significant advancements in wearable pressure sensors have been noticed over the course of the past several decades. These advancements have made it possible for these sensors to continuously and noninvasively monitor human physiological and pathological signals [14, 15]. The use of these biomedical indicators to properly assess cardiovascular conditions can then provide an individualized system of healthcare with better health outcomes, more convenient use, higher quality, and lower costs, all of which are crucial for dropping the prevalence and death rates of cardiovascular disease (CVD) [11, 13].

To collect cardiac-related physiological data, biomechanical sensing devices have been the subject of substantial research [12]. Using a stress or straining sensor attached to the vasculature or the skin near the arteries, biomechanical techniques can directly obtain information reflective of pressure changes in the vascular system [16]. This allows for the reflection of the pressure waves in the vasculature. In addition to this, they use a range of power that normally falls between nanowatts and microwatts, making them substantially more energy efficient. Additionally, since the waveform of the electrical signal recorded by mechanical sensors is identical to that of the arterial blood pressure, this relationship can be used to infer further data about the patient that is useful in the diagnosis of cardiovascular diseases. Therefore, the use of mechanical methods can offer a solution for long-term CVD pressure monitoring that is more accurate, more space efficient, and uses less energy [2, 11].

Recent research has shown that wearable sensors can be directly connected to the skin surface or integrated with clothes, which is more practical and has adequate patient compliance. These investigations were conducted by a number of different researchers. In contrast, implantable sensors necessitate surgical trauma, but they offer advantages that cannot be replicated for the detection of minute forces in deep tissues. The main problems that prevent clinical applications of implantable and wearable pressure devices for diagnostics are inaccuracies in measurement and a lack of long-term durability. Additionally, in order to obtain conformal contact with the surfaces of organs and soft tissues, the biocompatibility and flexibility of the sensors need to undergo further development [17–20].

Consequently, there is still a lot of room for growth and development in the use of biomechanical sensors for cardiac activity monitoring, notwithstanding recent optimization efforts in this area. Miniaturization in production and transmission via wireless means sensors can be implanted in the body in a less invasive fashion or worn on the body continuously to monitor a wide range of biomarkers and decrease in the need to recharge implantable sensors through the use of energy harvesting and conversion methods.

Diagnostic precision was increased with the use of multimodal signals such as microvascular flow; to address the demands of personalized medication and remote monitoring in healthcare, cutting-edge technologies like machine learning and cloud computing are being coupled with real-world physiological data to create novel monitoring systems. As a result of the efforts of scientists all around the world, there is hope that chronic illness monitoring in the cardiovascular system can be accomplished with the use of implantable and wearable sensors.

Latest developments in mechanical sensors for the cardiovascular system are summarized in this review. First, the characteristics and operation of four common mechanical sensors are outlined. After that, various techniques for keeping tabs on a patient's vital signs are covered, and details about the cardiovascular system's physiomechanical workings are provided. Furthermore, from an energy point of view, the potential application shift from battery-powered to self-powered sensors is considered. To wrap things up, a brief summary of why existing cardiovascular system monitoring needs to be improved is provided. The goal is to bridge the gap between mechanical sensing and the acquisition of physiological signals for the protection of cardiovascular health.

## 2. Cardiovascular System Biomechanical Activity

**Figure 1:**
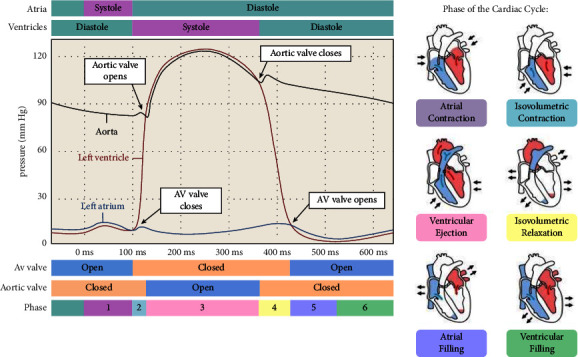
Blood pressure in the aorta compared to that in the ventricles and atria, as well as the rhythm of the heart.

There are arteries, capillaries, and veins that make up the cardiovascular system. The heart stands out among the body's organs as being unusually consistent in its form and function. The heart's powerful beating pumps enough blood to the body's tissues and organs to supply their metabolic demands. Because of this, the heart can be thought of as a series of unidirectional pumps. Based on this trait, the heart must generate adequate force to pump out blood it takes in; change the blood supply in response to the needs of the body pumped out per unit of time and guarantee a one-way flow devoid of reflux. The heart's physiological activity stems from its role as a biological pump. Reduced efficiency or, in the worst scenario, the full stoppage of physiological function will follow if any component of the pump is structurally weakened or malfunctions. The reasons of cardiovascular disease can be better understood with a more in-depth comprehension of the pumping function of the heart. Inner, middle, and exterior structures often make up a vascular wall, which is a tube that is hollow and has a structure that is made up of multiple layers of composite material that can bear the pressure of blood and is bound by extratubular tissues. Smooth muscle, elastic fibers, and collagen fibers are all the components of smooth muscle that are located in this layer, and they are largely responsible for the vessel's mechanical qualities. Vascular wall compliance is a key clinical measure of the elasticity or stiffness of the vascular system's cushioning function [21]. In general, the arterial vessel wall is more elastic and has a higher percentage of elastic fibers, the closer it is to the heart. When compared, the arteries further from the heart tend to be stiffer. Blood will flow through the arterial vasculature as pulse waves due to the heart's intermittent contraction and diastole [22]. As pressure waves, it is typically perceived. There are two main components to a pulse wave: the peak and the trough. During ventricular systole, the artery suddenly expands, as shown by the ascending branch, while during ventricular diastole, as seen by the descending branch, as shown in Figure 1.

## 3. The Principle of Cardiac Activity Sensing Systems

**Figure 2:**
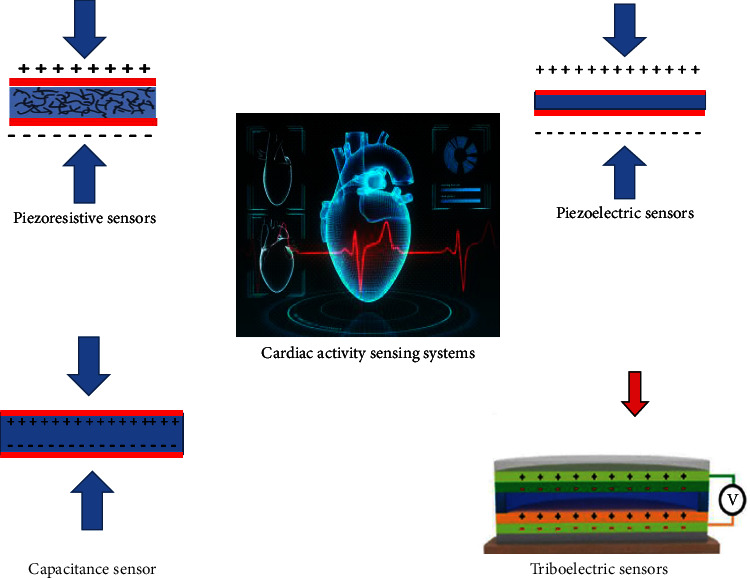
Schematic illustration of different sensing systems for cardiovascular monitoring.

Wearable or implanted strain sensors and pressure sensors are the primary stretchable sensors used in mechanical approaches for monitoring cardiovascular physiological parameters. These sensors rely on a variety of different fundamental sensing methods, including piezoresistive, piezoelectric, triboelectric, and capacitive [11, 23, 24], as shown in Figure 2.

The principle of the piezoresistive effect is at the core of the piezoresistive sensor. The piezoresistive effect describes the modification of the output electrical impulse caused by the pressure variation in the resistivity of pneumatically materials. Another technique of detection relies on the shift in electrical resistance brought about by architectural changes caused by increased pressure of dielectric materials. Piezoresistive impact sensors are powered by external pressures that bend the dielectric substantial, which in turn changes the distribution and contact state of the internal conductive filler and, consequently, the dielectric material's resistance [25].

Pressure sensors based on piezoresistive strain gauges have the benefit of being extremely durable. The consistency of both their functioning and their calibration may be relied upon over time. The straightforward design results in both lower costs and increased longevity. The sensors have a high level of durability, making them resistant to shock, vibration, and variations in dynamic pressure. Readout circuits are quite straightforward, which paves the way for high-resolution measurement. In most cases, the response time is less than one millisecond, and the output varies linearly with the pressure. They are capable of measuring pressures ranging from around 3 psi all the way up to about 20,000 psi; therefore, their applications are rather diverse (21 kPa to 150 MPa). Additionally, the output is consistent over time. There is the possibility of fusing the resistive elements to the diaphragm. This is a tried and tested method that has been put to good use for a considerable amount of time, but complications might arise when the adhesives are subjected to high temperatures or excessive pressure. A further possibility is to construct thin film resistors directly on the membrane itself. These are able to function well in temperatures that are higher and are better suited for use in severe environments [25].

Incorporating the piezoelectric phenomenon, piezoelectric sensors transform mechanical forces into electrical impulses. Piezoelectric are a type of noncentrosymmetric dielectrics. The electric fields imbalance between the upper and bottom electrodes is caused by the internal detachment of positive and negative charges caused by the material deformation that occurs under stress or strain. The pattern of the potential difference is supported by this mesoscopic dipolar in the crystal lattice, which is caused by the opposite movement of the positive and negative charge centres. As a result, the potential difference can be used as a proxy for atmospheric pressure [26].

Since piezoelectric materials convert mechanical energy into an electrical signal, they can be used to monitor a wide variety of physiological signals. They work particularly well for tracking variations in pressure over time, as many vital signs, such as the heart rate and breathing rate, are cyclical processes. Sound waves and tactile sensing both operate at pressures in the low pressure range (1 Pa–10 kPa). Intraocular pressure and cranial pressure are at the upper end of that spectrum. Blood pressure readings and some bodily motions fall into the range of systems with pressures 10–100 kPa. In order to achieve the desired pressure range and quality, piezoelectric sensors can be adapted structurally or materially [26].

By combining the triboelectric effect and electrostatic induction, a triboelectric sensor has the capacity to provide an electric signal to pressure stimulation. The triboelectric phenomenon happens when the surfaces of two materials with different triboelectric characteristics come into touch with each other and create different polarity. The magnitude of the cost is proportional to the dissimilarity in triboelectric characteristics of the two friction materials. Triboelectric characteristics generate a potential difference proportional to the quantity of opposing charge on each surface. Electrostatic induction causes inductive charges on the electrodes, which are generated by the applied pressure. Electrical output for sensing is generated when charge moves between electrodes and eventually reaches balance through an open load [27].

Triboelectric sensors, which can function as a self-powered device, are a rapidly developing field of technology. Triboelectric sensors are able to accurately monitor heart rates because of their excellent signal-to-noise ratios, pressure sensitivity, and high-voltage outputs achieved by turning mechanical energy into power. In addition to being lightweight because of their compact design, they also come in a wide range of materials from which to choose, including synthetic polymers, silicone rubber, silk, and cotton. Furthermore, triboelectric sensors are a practical everyday wearable gadget because of their diminutive size. Additionally, triboelectric sensors are rapidly developing with the purpose of gathering energy from human movement to power other devices. Several different types of wearable heart rate monitors based on triboelectric sensors have already been created [27].

In order to measure the felt mechanical load, capacitive sensors alter the capacitance value of the device and capacitance, in the context of a conventional parallel plate construction. The capacitance of a capacitor can be altered by applying a specific amount of force to its electrode plates. In light of this, pressure sensing is an achievable goal based on this approach [28].

As such, capacitive sensors use two electrodes and a compressible dielectric material to measure electrical fields. Applying force causes the gap to close and the capacitance to increase. In contrast to resistive technology, the two electrodes are separated at all times. Therefore, capacitive sensors can withstand repeated loads with minimal risk of damage.

Superior stability in terms of repeatability and longevity, as well as the ability to accurately monitor low pressure levels, are just two of the benefits of capacitive sensor technology over resistive. Integration of pressure (using arrays measuring thousands of points of force) and force sensors (measuring discrete points) with consumer electronics, robotics, ergonomics, medicine, and automobiles are only few of the uses being investigated [28].

## 4. Cardiovascular Physiological Parameters Monitoring

Despite the intricacy of cardiovascular physiology, the primary function of this system is quite straightforward, to supply oxygenated blood to critical organs and tissues. Important to this process is the heart's role as a pump. Everything that happens between the start of one ventricular systole and the start of the next is called “the cardiac cycle.” Consequently, the monitoring of the biomechanical processes are taking place in the cardiovascular system, such as blood pressure and endocardial pressure, pulse wave, and heart rhythm [29, 30].

### 4.1. Blood Pressure

Pressure exerted per unit area by blood against the vessel wall is what we call blood pressure. The three most important parameters in determining blood pressure are the amount of blood pressure, the pace at which blood is pumped into and out of the arteries, and the elasticity of arterial walls [21]. Systolic pressure is the pressure of blood as it is pumped out of the heart and into the arteries during the contraction segment of the rhythmic beating of the heart. Also, the pressure in the arteries is called diastolic when it is taken when the heart is at rest between beats [22].

Generally speaking, a person's blood pressure will be higher in the morning (or while awake) and lower during the night (or during sleep). Stroke, heart failure, and kidney failure are just some of the cardiovascular events that may be linked to a person's blood pressure readings before and after these two periods [31, 32]. Compared to single and ambulatory readings, continuous blood pressure is unique. For purposes of pacemaker adaptation, automatic examination of sleep disruption, diagnosis of heart problems, and cardiovascular evaluation, real-time arterial blood pressure variations with clinically significant waveforms and trends are of interest [31, 32]. There are a number of signals related to blood pressure, and their shapes all vary when there is a change in blood pressure during the hearts beat [21].

Heart and blood vessel hemodynamic are intrinsically linked to blood pressure. To a large extent, blood is turbulently viscous in the flexible capillaries (aorta); therefore, most of the energy transfer occurs along the vessel walls [30]. The pressure pulsations within the vessels are affected by both vasoconstriction and diastole, which have an effect on the blood volume. Auscultatory and oscillometric procedures are the standard in clinical blood pressure measurement. Absolute air pressure can be measured in two ways [21, 22]; both involve using an inflatable cuff in conjunction using a compressor and a pressure gauge for a gas. Taking a person's average BP reading requires only measuring their brachial blood pressure using a cuff, on the assumption that this reading faithfully reflects their central aortic pressure [21]. It is easy to use and does not require much maintenance, but it cannot be measured continuously. Injection of a pressure sensor into the aortic for the purpose of measuring blood pressure [31] is the most accurate method of measuring blood pressure at present because it enables monitoring of blood pressure in real time. Though, because of its invasive nature, this technique cannot be used for sustained observation. More mobility is afforded to the patient in the cuffless method of continuous blood pressure monitoring [32], which relies on a mechanical sensor.

Blood flow rate was calculated using a distinct arterial pressure tube and 17.8 mV/mmHg sensitivity, as reported by Ma et al. [33]; nevertheless, linearity is weak (*R*^2^ = 0.78). When adopting a core shell packing method, the monitoring capabilities remain at a high level for up to 72 hours after the chest has been sealed. The in vivo biocompatibility of the device is assessed after two weeks of implantation to prove its clinical efficacy.

Using helium plasma irradiation, Wei et al. [34] produced a molybdenum microstructured electrode, which they incorporated into a Ti3C2Tx nanosheet impregnated polyurethane sponge to create a piezoresistive pressure sensor. Pressure detection over a wide range (0–100 kPa) with a response time of 226 ms is possible, thanks to the electrode engineering strategy of a seamless transition between sponge deformation and MXene interlamellar displacement, yielding great sensitivity (1.52 mV kPa^−1^) and strong linearity (*R*^2^ = 0.9985). Furthermore, the hierarchical structures controlled by pore size, plasma bias, and MXene concentration are confirmed to play a vital role in enhancing the sensing performance through both experimental characterisation and finite element simulation. More evidence of the created flexible pressure sensor's potential in wearable biomonitoring and health evaluation is provided by its shown ability to measure human radial pulse, as well as to detect finger tapping, foot stomping, and object identification.

Using a porous dielectric layer (PDMSDIW), Kumar [35] demonstrated a relative change in capacitance of 0.07%–15% throughout an applied pressure range of 1 Pa to 100 kPa, with a sensitivity of 0.095 mV kPa^−1^. Over a wide range of external pressures, the device also shows increased sensitivity in comparison to an unstructured PDMS layer. The device's ultra-low detection limit of pressure is 1 Pa, and it displays a wide operating pressure range (1 Pa to 100 kPa), good working stability, quick reaction (110 ms), and high sensitivity. Also investigated was blood pressure monitoring, with tests showing that multiple oscillometric waveform signatures were produced by the devices for varying blood pressures. Thanks to its high performance characteristics, the flexible pressure sensor that was manufactured can be integrated into wearable blood pressure monitors and employed in biological settings.

Fang et al. [36] demonstrated a cheap, monitoring of pulse waveforms in high resolution and real time in moving and sweating situations, a lightweight, triboelectric detector made of a textile material that is functionally robust and that can convert minute skin deformations generated by artery pulsation into electrical energy has been developed. The accuracy of the textile triboelectric sensor, which is assisted by machine learning algorithms, was validated by comparison to the standard industry blood pressure gauge used in hospitals. Data driven cardiovascular diagnosis and simple data sharing are both possible with this approach.

A wireless, continuously operating blood pressure monitoring device was created by Yi et al. [37]. Integration, transition correction, and direct association using the kinetic model, the relationship between piezoelectric responsiveness and blood pressure, were determined by simulation and experimental analysis. Constant monitoring of blood pressure via a single, wearable piezoelectric sensor was shown to be possible without introducing motion artifacts. With these results, the debate over the piezoelectric response of pulse waves in the artery is settled, and the system is more convenient than those measured by the speed of pulse waves between different sensors. To help detect hypertension early and keep it under control on a regular basis, it is projected to be used to create a wearable, mobile blood pressure monitor.

Using self-powered ultrasensitive pulse sensors (SUPSs) located at several arterial pulse locations, Xu et al. [38] proposed a method for noninvasively monitoring many cardiovascular parameters. In order to calculate the arm fingertip PTT, we took the SUPSs' readings of pulse waveforms and found the offset between their peaks. Heart rate, pulse wave velocity, and blood pressure were all assessed and shown to be very compatible comparisons with results obtained with standard medical equipment when a linear fit is applied to the typical reading from an electronic cuff blood pressure monitor of the patient's blood pressure.

According to Meng et al. [39], a flexible weaving self-powered pressure sensor (WCSPS) has been developed. Its single-electrode working mode, ultrasensitivity of 45.7 mV Pa^−1^, and ultrafast response time of less than 5 ms are all made possible by plasma etching of PTFE to introduce surface nanowires. One hundred participants, ranging in age from 24 to 82 years and in varied states of health, had their real measurements used. The BP value was computed in real time by an enhanced genetic algorithm, and PTT was determined by subtracting the timestamps of the peaks in the fingertip and ear signals. WCSPS blood pressure readings varied by between 0.87 and 3.65 percent from those obtained using a commercial cuff-based device. All of these things prove that it is possible to have a real-time monitoring system for the cardiovascular system that is both effective and affordable.

Battery-free, wireless monitoring of arterial blood flow was recently reported by Boutry et al. [40]. The prepared pressure sensor is light and flexible, so it may be readily wrapped around arteries of any size. The fringe field capacitance technology it employs allows for both touch and noncontact arterial blood flow measurements. The sensor's low latency, quick response, great cycle stability, high durability, and straightforward, no disassembly installation make it ideal for minimizing vascular injury.

A wireless stent system incorporating soft sensors was created by Herbert et al. to suit insertion and manipulation needs in their vascular electronic system [41]. Two stretchy interconnect pressure sensors were attached to the stent in order to track changes in blood flow velocity in the artery, and a patterned dielectric layer was produced using aerosol jet printing to increase the capacitive sensor's sensitivity and response time. By using inductive coupling to enable wireless data transmission, the system is able to monitor hemodynamic in real time, including pressure, pulse rate, and flow. For the purpose of producing in situ polarized ferroelectric artificial arteries, Li et al. [42] devised an electric-field assisted 3D printing approach. These arteries can detect occlusions and measure blood pressure in real time without the need for batteries. Excellent piezoelectric characteristics (d33 > 12 pC N^−1^) were achieved through the combined efforts of potassium sodium niobate (KNN) particles and a polyvinylidene fluoride (PVDF) polymer matrix. Due to the sinusoidal structure and 3D printing of the ferroelectric material, the mechanical modulus is close to the vascular level, enabling sensitive sensing of pressure changes (0.306 mV mmHg^−1^, *R*^2^ > 0.99). (11.25–225.00 mmHg). Sensitive pressure sensors can identify even the smallest changes in blood vessel motion, allowing for early diagnosis of partial occlusions (like thrombosis) and reducing the risk of implant failure.

To monitor blood pressure, Cheng et al. [43] used a piezoelectric thin film (PETF) to create an implanted device that requires no external power source. The sensitivity of the sensor was 14.32 mV mmHg^−1^, and its output voltage correlated well with aortic systolic blood pressure (*R*^2^ = 0.971). Yorkshire swine adults were implanted with the PETF to monitor their blood pressure and provide a visual alert if they were showing signs of hypertension in real time. For further development of both implanted and external medical devices, the manufacture of electromechanical coupling biopolymers is essential. The first portable thrombus detector was announced in 2019 by Li's team [44]. Organic piezoelectric nanofibers (OPNs) with enhanced piezoelectricity, fatigue resistance, stability, and biocompatibility were prepared by combining polyvinylidene fluoride (PVDF) with hydroxylamine hydrochloride (HHE) in a core/shell structure. So, a soft sensor made of PVDF/HHE OPNs was created to detect and measure even the most minute pressure variations in vivo. When implanted in pigs, it demonstrated extremely high detection sensitivity and accuracy and was able to record micropressure changes outside the cardiovascular wall. Changes in elastic properties of the cardiovascular system, the start of atrioventricular heart block, and thrombus formation can all be reflected and differentiated in the output piezoelectric signal in real time. This biological data can aid in the evaluation and diagnosis of thrombosis and atherosclerosis, particularly in cases of recurrent deep thrombosis after surgery. One-step preparation of core/shell PVDF/dopamine (DA) nanofibers (NFs) with high beta phase content and self-aligned polarization features was reported by Li et al. in 2021 [45]. Solid intermolecular contact between the -NH2 group on DA and the -CF^2^ group on PVDF improves polymer chain alignment and facilitates phase nucleation, which is thought to be the key to the development and alignment of the phase PVDF, which has a self-assembled core/shell structure. The produced PVDF/DA NFs are very stable, biocompatible, and display improved piezoelectric characteristics. A highly sensitive and accurate all-fiber soft sensor capable of picking up the minute mechanical forces produced by the diaphragm's movement and the heart's pulsing of blood was developed from this research. This means it can be used to screen for and detect respiratory and cardiovascular disorders in their earliest stages, allowing for earlier intervention and better outcomes. 12 × Biosensors, 2022 review by peers alignment of polymer chains and phase nucleation are both improved by the -NH_2_ group on DA and the -CF_2_ group on PVDF, which appears on atomic position. The produced PVDF/DA NFs are very stable, biocompatible, and display improved piezoelectric characteristics. A highly sensitive and accurate all-fiber soft sensor capable of picking up the minute mechanical forces produced by the diaphragm's movement and the heart's pulsing of blood was developed from this research. Therefore, it can be used for early detection of cardiovascular and respiratory disorders and risk assessment.

As it happens, different types of self-powered stent ideas based on the triboelectric nanogenerator (TENG) have been developed. A self-sensing, biocompatible, and nontoxic stent made from composite mechanical metamaterials was proposed by Barri et al. [46]. This stent would be self-powered by harvesting energy from artery pulsations. A commercial balloon dilator catheter can be used to deploy the stent while continually monitoring local hemodynamic changes for signs of tissue overgrowth or arterial restenosis. The design presented here is merely a proof of concept; it is not optimized for maximum efficiency or durability. However, implantable stents can continuously assess applied forces and harvest energy from external mechanical excitation if they are designed with the concept of self-powering in mind. It demonstrates a viable technique for introducing a degree of flexibility into intelligent healthcare systems in order to accommodate virtual living mechanisms, and it is crucial for building cutting-edge medical stents. Using the triboelectric effect between bioabsorbable materials, Ouyang et al. [47] reported in 2021 a pressure sensor that can immediately transform pressure changes in the environment into electrical data. The linearity (*R*^2^ = 0.993), sensitivity (11 mV mmHg^−1^), and endurance of this bioabsorbable triboelectric sensor (BTS) were all above average (450,000 cycles). It has a high efficiency (59.54%) and a service life of 5 days after implantation in large animals, during which it can detect aberrant vascular occlusion occurrences. The various sensing system for blood pressure monitoring are given in Table 1.

### 4.2. Pulse Wave and Cardiac Output

By virtue of its high sensitivity and fast response time, the mechanical pressure sensor is better as a means of identifying the pulse wave's accompanying pressure signal and subsequently obtaining a more reliable reading of that signal. The elastic connective tissue and muscle that make up arteries form tubes. A pulse signal is generated when the ventricle contracts and relaxes at regular intervals during cardiac cycles [48].

The pulse rate is directly proportional to the rate of the heart's electrical activity and is hence typically measured in beats per minute (b.p.m.). Both optical and capacitive pulse pressure sensors are utilized in the present day's wearable healthcare systems [12]. One is a battery-operated pulse sensor, which includes piezoresistive, capacitive, and optical sensors. On the other hand, there are pulse sensors that do not need external power, such as piezoelectric and triboelectric pressure sensors.

Lin et al. [49] proposed an iontronic pressure sensor that is both sensitive and adaptable; it has a linear sensitivity of 13.5 mV kPa^−1^, a fast reaction time, and outstanding stability over 5000 loading/unloading cycles. This sensor provides for high-resolution, steady pulse waveform detection regardless of finger mobility. The analysis of collected fingertip pulse waveforms from people of varying ages, health statuses, and sex indicates that fingertip pulse information is very similar to that obtained from the radial artery. This research provides support for the hypothesis that the fingertip is a suitable platform for detecting pulse signals, offering a feasible replacement for costly and complicated health monitoring solutions.

Capacitive pressure sensors based on a porous structure of polydimethylsiloxane (PDMS) thin film have been proposed by Kang et al. [50], who drew inspiration from the multilayered porous structures found in mushrooms, diatoms, and *Spongia officinalis*. With the use of a bioinspired porous dielectric layer, we are able to create high performance pressure sensors with a sensitivity of 0.63 mV kPa^−1^, a stability of 10,000 cycles, a reaction time of 100 ms, a relaxation time of 100 ms, and a detection threshold of 2.42 Pa. It is also shown that the resulting pressure sensors may be used to create multipixel arrays, leading to accurate tactile detection of different touch shapes in real time. The developed high performance flexible pressure sensors could pave the way for novel uses in cutting-edge HMI (human machine interface) systems, RS (robotic sensory) systems, and WHM (wearable medical monitoring) devices.

The nanohemispherical pressure sensor developed by Meng et al. [51] *p* is able to monitor fingertip pulse by being mounted to the surface of commonly used items. An ultrasensitive 49.8 mV/Pa was achieved by the compact sensor, together with a remarkable reaction time of less than 6 ms and a long-term durability of over 4 months. For example, the sensor has been shown to be useful for measuring fingertip pulse waves and extracting distinctive points of the waveform from the surfaces of keyboards, mobile phones, and the human skin. Since this sensor performs so well, we are able to create a wireless system for monitoring arteriosclerosis in real time. Diagnosis of antidiastole of arteriosclerosis was aided by evaluating the defining features of the pulse waveforms recorded from 54 healthy volunteers. The suggested sensor is anticipated to be a cost-effective replacement for existing complex medical equipment and to find widespread application in wireless cardiovascular monitoring systems.

**Figure 3:**
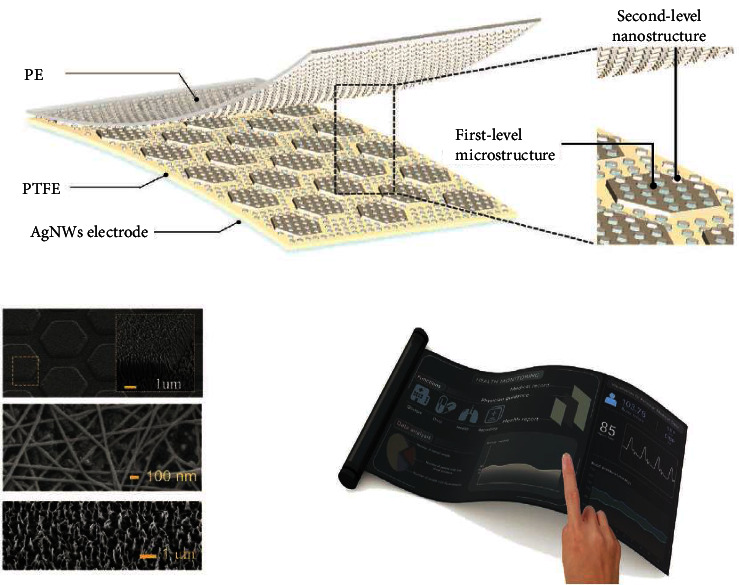
The ultrathin and flexible sensor (UFS) shown in a simplified schematic form, along with its single-electrode mode application for measuring when fingers are touched to a surface.

To keep tabs on vitals like HR and BP in real time, Fan et al. [52] designed a liquid capsule pressure sensor (LCPS) that could be worn. A liquid capsule has a filling fluid and a flexible membrane. The pressure sensor system's alignment accuracy with the artery was significantly loosened to around 8.5 mm by sending the pulsation signal detected by the capsule to an implanted piezoresistive sensor in accordance with Pascal's principle. The mean correlation coefficient between the LCPS derived heart rate and the ECG derived heart rate for the 11 subjects (i.e., those wearing the LCPS without position calibration) is 0.9944. The subject's systolic and diastolic beat to beat BP values were calculated using the ECG, the PPG, and the LCPS and then correlated to blood pressure readings recorded using cuff devices. The findings show that the LCPS/ECG duo is just as reliable as the standard PPG/ECG combination. In addition to ensuring the sensor's high fidelity epidermal pulse signal, this low-power (35 nW) LCPS is also spatially insensitive, making it simple for the user to install. Pulse waveform optimization can also be achieved by measuring temporal and spatial pulse waves with a wearable pressure sensor array [53]. Thin film transistor (TFT) arrays combined with thin sheets of very sensitive piezoresistive sensors were fabricated by Baek et al. using the inkjet printing technique [53]. Strategic modulation of the TFT operating voltage increased pressure sensitivity (16.8 mV kPa^−1^) while simultaneously reducing power usage (101 nW). An ultrathin pressure sensor array, consisting of 100 pressure detecting pixels, was worn to generate a two dimensional distribution of pulse waves throughout the wrist. In order to construct a space time pulse wave map that demonstrates the position dependency of pulse amplitude, it is able to precisely measure both the pressure signal and the artery's position. Therefore, the augmentation index, a metric for gauging arterial stiffness, can be properly extracted by pinpointing arterial lines. Flexible and sensitive pressure sensors were created by Su's team of researchers [54]. This sensor's high quality sensing performance held up even after being stretched. Through the integration of a mechanical multilayer micropyramid structure and an ionic impedance sensing element in a cooperative design, we were able to reduce the lowest detectable pressure to 0.72 Pa while maintaining a strain insensitivity of 98% at 50% tensile strain. This flexible pressure sensor can be attached to a pneumatically powered soft manipulator, allowing for remote physical diagnosis and treatment of patients. Increased development of quantitative stress resulting on flexible interfaces and high-accuracy sensory tracking on stretch interfaces will substantially benefit future improvements in the identification of skin interactions with humans or soft machines in a reliable manner. Park et al. [55] used Pb[Zr_x_Ti_1-x_]O_3_ (PZT) to create an ultrathin conformal piezoelectric sensor that allows for conformal contact between the sensor and skin. Coating high quality PZT films onto a sapphire substrate, annealing the substrate, and then peeling off the PZT films to transfer them to an ultrathin polyethylene terephthalate (PET) substrate (4.8 m) is a common technique for creating thin, flexible electronics. Afterwards, gold forked finger electrodes were made and placed on top of the thin PZT substrate. Bandaging the wrist held the piezoelectric sensor that wirelessly transmitted the measured pulse signal to a nearby smartphone. With a friction layer made of nanostructured metallic copper and polymer film and an encapsulating layer of flexible polydimethylsiloxane (PDMS), Ouyang et al. [56] developed an autocharging ultrasensitive pulse sensor (SUPS). The self-activating, ultrasensitive pulse sensor outputs an electrical signal that may be transferred through Bluetooth without the need for signal amplification, allowing for the early detection and diagnosis of cardiovascular disease. The ultrathin and flexible sensor (UFS) created by Wang et al. [57] is made up of a polytetrafluoroethylene (PTFE) layer, a polyethylene (PE) friction layer, and an AgNWs electrode layer (Figure 3). A multilayer microstructure was used to alter the PTFE surface; the first layer was a hexagonal microstructure, and the second layer was nanowires. The results showed that the multilayer microstructured PTFE exhibited a sensitivity of 7.2 V kPa^−1^ and a response time of less than 4 ms, with an output voltage 7 and 2.7 times higher than the nonmicrostructured and single nanowire microstructured PTFE, respectively. At a static force of 1500 mN, the sensitivity was still up to 0.15 mV Pa^−1^. The UFS's superior qualities allow it to pick up on pulse pulses as tiny as those coming from a fingertip. What is more, by incorporating the UFS into mobile devices, this study has effectively provided a proof of concept for a healthcare system.

Central to the monitoring of hemodynamically unstable patients in intensive care and surgical settings is cardiac output (CO), the volume of blood evacuated per minute from one ventricle. Some of these techniques are the clinical gold standard, intermittent pulmonary thermodilution using a pulmonary artery catheter, while others, such as ultrasonography Doppler, bioimpedance, and pulse wave analysis, are less intrusive. Patients who are hemodynamically unstable are closely monitored in the operating room and intensive care unit by measuring the volume of blood drained from a single ventricle per minute. Invasive periodic pulmonary thermodilution with a pulmonary artery catheter, noninvasive and minimally invasive ultrasonography with Doppler, and pulse wave modelling are all methods that can be used to assess cardiac output [47, 58].

The pulmonary thermodilution method of CO monitoring is the gold standard in clinical practice since it allows for a full evaluation of cardiovascular function. However, noncardiac surgery patients with low or intermediate risk are rarely good candidates for this procedure. Transthoracic echocardiography (TTE) using ultrasound Doppler technology is now a standard monitor in cardiac operating rooms and intensive care units. TTE is a well-established method of clinical hemodynamic assessment. However, there are a few restrictions, such as the need for an appropriate ultrasound window, the reliance on an operator, and the moderately real-time monitoring. Contrarily, pulse wave analysis enables constant, real-time monitoring of CO variations. Additionally, it can be utilized for goal directed hemodynamic therapy during the perioperative period. All pulse wave monitoring techniques start with the assumption that the pressure wave has a known and consistent relationship with the measured quantity [59]. Since the pressure wave is the end result of the interaction between the heart's expelled blood and the artery system, the harmonic information included within the wave itself is highly correlated with the typical parameter changes in the cardiovascular system. Stroke volume (SV) is the volume of blood discharged from the left ventricle during a cardiac cycle, and cardiac output (CO) is the product of SV and heart rate (HR). Calibration is used to rectify the pressure volume connection for a more precise estimate of SV when employing a pulse wave-based monitoring approach for the arterial system. Tonometry of the radial artery, a noninvasive method of measuring blood pressure, allows for continuous waveform monitoring of arterial blood pressure [60] and is a feature of the DMP Life system (DAEYOMEDI Co., Ansan si, South Korea). A computer program was used to calculate CO by analysing the systolic segment of the arterial blood pressure waveform and factoring in the patient's demographic and biometric information. Hemodynamic data were taken at random from the radial arteries of 107 individuals undergoing preoperative cardiothoracic surgery. Testing and correlating were done using the Bland Altman method and the Pearson correlation. This study's findings corroborate the accuracy and consistency of the blood pressure pulse analysis method with that of the ultrasound Doppler method in determining SV and CO. Movements of the patient's limbs, whether active or passive, might degrade the quality of the arterial blood pressure waveform, rendering the pulse wave analysis inaccurate [47]. Despite the fact that these electromechanical sensors are not ideal for CO tracking has lagged behind the rapid technological advancements in the monitoring of pulse waves and blood pressure, it remains an attractive choice for cardiovascular monitoring in clinical surgery and critical care patients. The various sensing system for pulse wave monitoring is given in Table 2.

### 4.3. Endocardial Pressure and Heart Rate

Pacemakers, cardiac resynchronization treatment (CRT) devices, and defibrillators are all examples of cardiovascular implanted electronic devices (CIEDs) that play an important role in decreasing disease and death by observing, restoring, and controlling heart function [13, 61]. Endocardial pressure (EP), including atrial and ventricular pressure, is a crucial sign for assessing the heart's pumping capacity [62]. Clinically, the only reliable approach to gather EP data is by the use of a piezoresistive pressure sensor for intracardiac catheters; however, this method is invasive to implement and cannot collect data continuously over extended periods of time [13]. The cardiac catheter's external recorder is cumbersome and can be a source of operational difficulties and low patient compliance [63]. The intermittent nature of EP monitoring in clinical practice raises the possibility of missing transient/silent symptoms, hence leading to misdiagnosis of patients [64].

Improvements in real-time medical monitoring will come from self-powered sensors that require little to no external power but still have good performance while monitoring physiological signals (for active endocardial monitoring, for instance). Long term in vivo cardiac implant safety was assessed by Li et al. using an implantable nanogenerators (i-NG) system made of soft and flexible nanogenerators [65]. Pigs had a system including piezoelectric PVDF-basedi-NG, leads, and receivers implanted in their epicardial membrane for a total of two months. During diastole, as the heart muscle relaxes and expands, the PVDF membrane stretches outward and creates a voltage difference. When ischemia was induced by temporarily blocking the left anterior descending (LAD) artery, the i-NG showed a rapid increase in peak to peak voltage (Vpp) from 2.3 V to 4.5 V, which is clinically indicative of a heart attack. Ma et al. [33] introduced a self-powered, bendable, implantable triboelectric active sensor (iTEAS) for continuous monitoring of several physiological and pathological indicators. Once the sensor has been appropriately implanted onto the surface of the heart, the triboelectric layer will separate and make contact with the heart during systole and diastole, coupling contact electrification and electrostatic induction to continually output electrical signals. When the chest was closed for 72 hours, there was no change in the monitoring function. With a 99% degree of precision, the device can track heart rate. Rhythm disorders such as atrial fibrillation, ventricular tachycardia, and premature ventricular beats can be identified in real time. After two weeks of implantation, the gadget was biocompatible enough to be used clinically. There is significant potential for the suggested iTEAS to be used in healthcare as a multipurpose biological monitor that does not require an external power supply.

In patients with heart failure and reduced cardiac function, monitoring changes in endocardial pressure (EP) by a minimally invasive method is of critical clinical importance. For continuous EP monitoring, Liu et al. [64] presented a triboelectric based, miniature, flexible, ultrasensitive, and self-powered endocardial pressure sensor (SEPS). When placed in an in vitro fluid pressure simulator, the pressure signals acquired using commercial probes follow the same pattern as the SEPS. The in vivo monitoring performance of the SEPS was validated by inserting minimally invasive catheters into the left atrium of mature male Yorkshire pigs. Blood pressure was taken from the left femoral artery through an arterial pressure catheter (FAP). The SEPS voltage output was measured independently throughout systole and diastole of the heart, and it almost exactly matched the FAP observed during adrenaline controlled systole. The device oscillated more than 100 million times and was mechanically and electrically stable throughout in vitro testing. Moreover, there was a negligible danger of hemolysis and coagulation cascade activation due to the encapsulating layer's haemocompatibility. The field of implanted health monitoring stands to benefit greatly from these developments, which contribute to the safe sensing of pressure situations, diagnosis, and monitoring of cardiovascular disorders.

**Figure 4:**
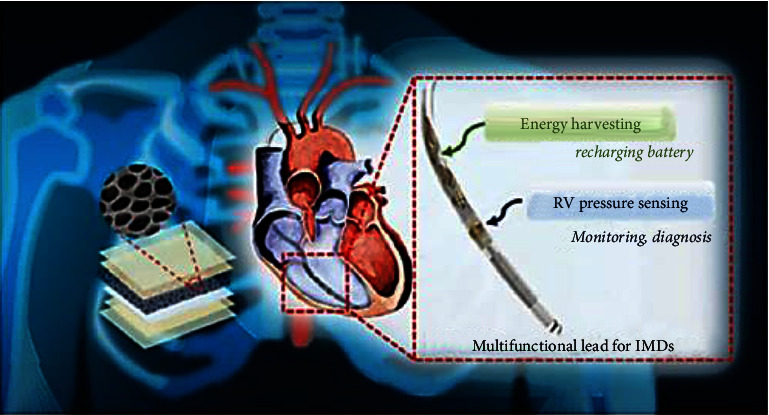
Multipurpose pacemaker lead that integrates energy harvesting and sensing techniques.

Part of the next frontier in medicine is the use of implantable medical electronics that can both monitor and treat disease. Dong et al. [66] suggested a method that combined energy harvesting from the heart with monitoring of endocardial pressure. Cutting-edge piezoelectric porosity liquid crystal energy storage devices can convert the rotational power induced by heart systole and diastole into electric power. As a potential early sign of arrhythmia, variations in right ventricular output can be tracked and recorded. Battery life is extended by 20% and power consumption is reduced when conjunction with the wires of preexisting pacemakers and implantable cardioverter defibrillators (Figure 4).

Li et al. [67] described a way for immediately charging a pacemaker that can regulate pig cardiac in vivo by utilizing the energy from the pulse without the requirement for any extra energy vital capacity. Because of its elastic spine and two piezoelectric composites, the generator is able to produce a high in vivo output current of 15 A. This research can aid in two areas: the creation of self-powered pacemakers and the implementation of piezoelectric harvesting technologies to solve the problem of powering implantable medical equipment (Figure 4). Ouyang et al. [68] demonstrated the feasibility of a symbiotic pacemaker based on implantable TENG for energy harvesting, storage, and cardiac pacing in a large animal model. The open circuit voltage of an implanted TENG might reach as high as 65.2%. On average, 0.495 J of energy was extracted from each cardiac cycle, which is more than the minimum required for endocardial pacing (0.377 J). The symbiotic pacemaker was successful in treating sinus arrhythmias and halting the progression of the disease. Because of its supercapacitors, sufficient endurance, and outstanding coefficient of performance, these TENGs are likely to find usage in diagnostic and treatment uses, including in vivo synergistic biosensors. The various sensing system for endocardial pressure and heart rate monitoring are is in Table 3.

## 5. Conclusions and Outlook Trends

Pulse waves, blood and endocardial pressure, and heart rhythms are the primary uses of mechanical sensors that hold improvement for use in evaluating cardiovascular health. In conclusion, mechanical sensors have the potential to improve cardiovascular health monitoring. Depending on the application, wearable sensors can be sewn into garments or adhered to the skin itself. The second option is preferable due to the fact that it is more practical and has sufficient patient compliance. Implantable sensors, on the other hand, require surgical trauma in order to be placed, but they offer benefits that cannot be imitated and are particularly useful for the detection of minute forces in deep tissues. Implantable and wearable pressure devices that are employed in diagnostics face the primary issues of ensuring accurate measurement and maintaining their functionality over an extended period of time. These are the obstacles that must be overcome before these gadgets can be used in clinical settings. In addition, there is a requirement for further improvements to be made to the sensors' adaptability and cytocompatibility in order to accomplish conformal contact with the surfaces of soft tissues and organs. As a consequence of this, the use of biomechanical sensors in the monitoring of CVD still faces a great deal of difficulties and a substantial amount of opportunity for development, and there is a tendency toward optimization.

A cardiac monitoring system needs to be able to function for many years in a highly dynamic environment, where it is constantly deformed and undeformed, contacted and uncontacted, in order to be therapeutically transferable.

It is crucial to think about mechanical biocompatibility; some designs are not healthy for the human body because they put too much pressure on the organs or the musculoskeletal system. This is especially crucial for heart adjacent devices; therefore, it is common practice to test them in animal models using no power to ensure they do not interfere with operation. Analysing the adhesion and delamination of the electrodes, as well as the responses between the materials in the body and the components of the cardiac monitoring system, are all areas that need more study. Taking into account a wide variety of drive frequencies, including frequent changes between low and high, is particularly important for cardiac sensing devices to maintain reliable operation.

One of the key drivers behind the advancement of the cardiac monitoring system is the provision of sustainable energy to construct implantable medical devices that can be used for a lifetime. Since the device will be implanted and remain in the body for years or perhaps decades, it must pass stringent in vivo evaluation requirements for biocompatibility, implantation method and site, and long-term endurance. As it stands, there are no unified evaluation criteria or methodology in place, and these assessments are neither systematic nor comprehensive. The surgical technique must be standardized appropriately. There are several potential locations for implanting a device; they include the heart, lungs, diaphragm, and aorta; therefore, it is important to establish the optimum implantation site and fixation approach.

Using energy collecting and transformation systems, we can achieve the autocharging detecting system, which significantly decreases the required charging time for implantable sensors. One of two targets, low energy sensing or self-powered sensing, will get the job done. A more complete extraction of information from multimodal signals, such as microvascular flow, is necessary for the continuous monitoring of several physiological parameters. The efforts of researchers from all over the world have led to the development of sensors that can either be worn or implanted. These sensors show a great deal of promise as potential tools for the continuous monitoring of cardiovascular disease and have been the result of the efforts of researchers from all over the world.  Table 4 presents advantages and disadvantages of capacitive, piezoresistive, and piezoelectric triboelectric sensors.

## Figures and Tables

**Table 1:** Representative sensing system for blood pressure monitoring.

Sensing mechanism	Sensitivity	Reference
Triboelectric	17.8 mV/mmHg^−1^	[33]
Piezoresistive	1.52 mV kPa^−1^	[34]
Capacitive	0.095 mV kPa^−1^	[35]
Triboelectric	0.21 *µ*A kPa^−1^	[36]
Piezoelectric	20 mV mmHg^−1^	[37]
Triboelectric	10.29 nA kPa^−1^	[38]
Triboelectric	45.7 mV Pa^−1^	[39]
Piezoelectric	0.306 mV mmHg^−1^	[44]
Piezoelectric	14.32 mV mmHg^−1^	[45]
Piezoelectric	16.2 mV Pa^−1^	[42]
Triboelectric	11 mV mmHg^−1^	[47]

**Table 2:** Representative sensing system for pulse wave monitoring.

Sensing mechanism	Performance	Reference
Capacitive	13.5 mV kPa^−1^	[49]
Capacitive	0.63 mV kPa^−1^	[50]
Capacitive	49.8 mV Pa^−1^	[51]
Piezoresistive	4.2 mV kPa^−1^	[52]
Piezoresistive	16.8 mV kPa^−1^	[53]
Piezoelectric	18 mV kPa^−1^	[54]
Triboelectric	7.2 V kPa^−1^	[55]
Piezoresistive	0.15 mV Pa^−1^	[56]

**Table 3:** Representative sensing system for endocardial pressure and heart rate monitoring.

Sensing mechanism	Performance	Reference
Triboelectric	2.8 V mmHg^−1^	[65]
Triboelectric	12 *μ*A kPa^−1^	[33]
Triboelectric	1.195 mV mmHg^−1^	[64]
Piezoelectric	15 *μ*A kPa^−1^	[68]
Triboelectric	65.2 mV mmHg^−1^	[67]

**Table 4:** Benefits and drawbacks of piezoelectric, piezoresistive, and capacitive triboelectric sensors.

Sensor type	Benefits	Drawbacks
Piezoelectric	Wide frequency response range High sensitivity Simple structure Cost effective	Dynamic signal only Leakage of electrical charge

Piezoresistive	Wide response range Simple structure High resolution	High temperature errors Complex manufacturing process

Capacitive	High stability Good dynamic response	High impedance Poor load capacity

Triboelectric	High sensitivity Simple structure Cost effective	High impedance Dynamic signal only

## Data Availability

The data used to support the findings of this study are available from the corresponding author upon request.
